# A gravitational contribution to perceived body weight

**DOI:** 10.1038/s41598-019-47663-x

**Published:** 2019-08-07

**Authors:** E. R. Ferrè, T. Frett, P. Haggard, M. R. Longo

**Affiliations:** 1 Department of Psychology, Royal Holloway, University of London, London, UK; 2 Deutsches Zentrum für Luft- und Raumfahrt e.V. (DLR), Cologne, Germany; 3 Institute of Cognitive Neuroscience, University College London, London, UK; 4 Department of Psychological Sciences, Birkbeck, University of London, London, UK

**Keywords:** Cognitive neuroscience, Human behaviour

## Abstract

The weightlessness experienced by astronauts has fascinated scientists and the public. On Earth, body weight is given by Newton’s laws as mass times gravitational acceleration. That is, an object’s weight is determined by the pull of gravity on it. We hypothesised that perceived body weight is – like actual weight – dependent on the strength of gravity. If so, changes in the experienced strength of gravity should alter the experience of one’s own body weight. We asked participants to estimate the weight of two body parts, their hand or their head, both in normal terrestrial gravity (1 g) and during exposure to experimentally altered gravitational fields, 0 g and +1.8 g during parabolic flight and +1 g using a short arm human centrifuge. For both body parts, there was an increase in perceived weight during the experience of hypergravity, and a decrease during the experience of microgravity. Our results show that experimental alterations of gravity produce rapid changes in the perceived weight of specific individual body parts. Traditionally, research has focused on the social factors for weight perception, as in the putative role of mass media in eating disorders. Our results, in contrast, emphasize that the perception of body weight is highly malleable, and shaped by immediate sensory signals.

## Introduction

Returning to Earth from the International Space Station, Canadian astronaut Chris Hadfield was struck by the sudden re-experience of his body’s weight. “Right after I landed”, Hadfield observed, “I could feel the weight of my lips and tongue”^1^. Previous research on the perception of body weight has focused on body satisfaction, body image and risk for eating disorders^2–4^. Hadfield’s observation, however, emphasizes the fact that the body, like any object, has weight only with respect to a gravitational field. The role of gravity in shaping perceptions of body weight has rarely been studied.

All objects are characterized by physical properties such as volume, density, and mass. While we do not usually conceive of our body as a physical object, it has the same properties as any other object. On Earth, the weight of the body (like any other object) is given by Newton’s laws as mass times gravitational acceleration (w = m * g). That is, the weight of an object is determined by the pull of gravity on it. Thus, an object’s physical weight may change from place to place: if a body weighs 54 kg on Earth, its weight will be just over 9 kg on the Moon, since the Earth’s gravitational force is six times stronger than the Moon’s. Previous research has investigated the extent to which the brain has internalized physical laws including laws of motion^5^ and gravity^6^. Remarkably, however, virtually no research has investigated how sensory signals specifying gravity modulate the *perceived* weight of body parts. Although it might seem trivial to show that perceived weight changes together with actual weight, it is important to identify on what sensory input these perceptual estimates are based. Reports from astronauts, such as Hadfield’s quote above, have provided anecdotal suggestions that a sudden change in the physical strength of gravity may produce rapid modulations of the perceived weight of body parts.

No single sensory signal informs the brain about the weight of body parts. However, sophisticated organs in the inner ear, the vestibular receptors, provide a reference for gravitational processing. The vestibular system comprises the semi-circular canals (anterior, posterior, and horizontal) which detect angular rotations of the head around three cardinal axes (yaw, roll, pitch), and the otolith organs (utricle and saccule) which detect linear acceleration and gravity. On Earth, when the head moves with respect to gravitational acceleration, the otoliths shift with the direction of gravity, moving the hair cells receptors and signalling to the brain head position relative to gravity. Thus, when the vestibular system works efficiently, the pull of gravity generates a constant sensory flow from early fetal life until death. These vestibular-gravitational signals are integrated with sensory signals from vision, proprioception and viscera. Consistent with this view, vestibular inputs do not project to any primary unimodal brain areas. Rather neuroimaging studies have identified a distributed network of brain regions including the posterior and anterior insula, temporoparietal junction, superior temporal gyrus, inferior parietal lobule, and somatosensory cortices^7^.

Here, we show that experimental alterations of the strength of physical gravity induced by parabolic flight and with a ground-based human short arm centrifuge produce rapid changes in the perceived weight of individual body parts. We asked participants to estimate the weight of two body parts, their right dominant hand or their head, both in normal terrestrial gravity (1 g) and during exposure to experimentally altered gravitational fields, 0 g and +1.8 g during parabolic flight and +1 g using a short arm human centrifuge. For both body parts, there was a clear increase in perceived weight during experience of hypergravity, and a decrease during experience of microgravity. Our results demonstrate that the brain performs an on-line computation of gravitational strength in order to construct a perception of one’s own body weight. This core aspect of body image and self-awareness therefore has a direct, sensory origin. This implies that perceived body weight is highly malleable, and reflects interpretation of immediate sensory inputs, rather than only stored representations or semantic beliefs about the body. Overall, our work identifies a key role of gravity in shaping bodily experience, potentially suggesting new approaches to understanding conditions such as obesity and eating disorders.

## Results

Participants were asked to make absolute verbal judgements (in grams) of the perceived weight of their dominant right hand and their head during a European Space Agency (ESA) parabolic flight campaign on board the Airbus Zero-G aircraft (Airbus A310). Parabolic flight generates alternating periods of hypergravity (+1.8 g) and microgravity (0 g), each lasting approximately 20 sec. Baseline measures in terrestrial gravity (1 g) were performed on board the aircraft before and after the flight. The participant’s head and hand were resting comfortably and movements were not allowed. This means that potential changes in perceived weight cannot be due to experienced differences in the amount of effort required to move body parts, but must be due to changes in the perception of gravity. By measuring perceived body-part weight in these altered gravity conditions, we aimed to identify whether perceived weight adjusts to sudden changes in the experienced strength of gravity.

Participants’ actual body weight (*M*: 79 ± 2.6 kg) was obtained with a high-precision scale immediately before the flight. Each participant’s hand and head weight were estimated using established norms for hand and head weight as a proportion of total body weight^8,9^. The actual weight of the hand and the head were estimated as 0.66% and 7.30% of total body weight, respectively. On average, the estimated weight of the hand was 521.4 ± 17.4 g and the weight of the head was 5767.0 ± 193.1 g.

**Figure 1:**
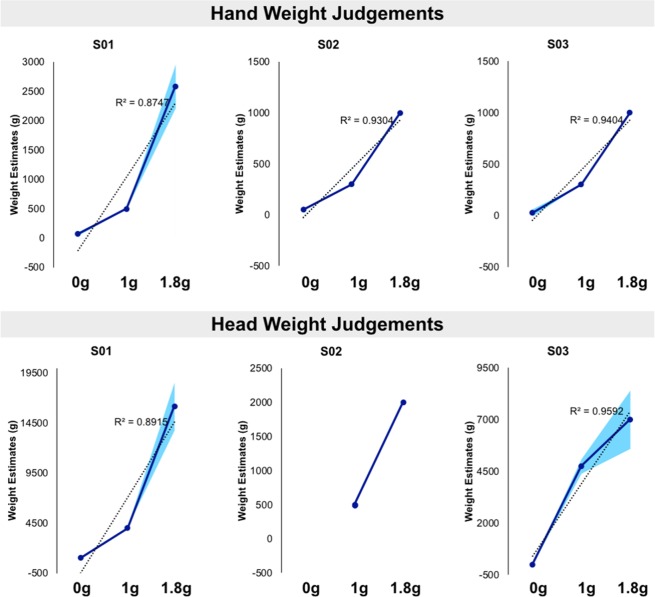
Parabolic flight: Body weight estimates results. The experiment took place during a parabolic flight campaign on board the Airbus Zero-G aircraft (Airbus A310). Parabolic flight generates alternating periods of hypergravity (+1.8 g) and microgravity (0 g), each lasting approximately 20 sec. Baseline measures in terrestrial gravity (1 g) were performed on board the aircraft before and after the flight. Participants made absolute judgements in grams of the perceived weight of their own right dominant hand and head. Individual data are presented for both body parts and in function of the strength of physical gravity (0 g, 1 g, 1.8 g). Compared to terrestrial gravity, both hand and head weight estimates were reduced in microgravity and increased in hypergravity. Blue lines indicate individual averages and coloured shades represent standard deviations. Dotted lines indicate the linear fitting.

Changes in the physical strength of gravity altered perceived body weight, as seen in Figure 1. Compared to terrestrial gravity (perceived hand weight: 380.1 ± 109.5 g; perceived head weight: 3600.0 ± 1781.9 g), both hand and head estimates were reduced in microgravity (perceived hand weight: 55.6 ± 30.1 g; perceived head weight: 750.0 ± 462.9 g) and increased in hypergravity (perceived hand weight: 2055.6 ± 845.7 g; perceived head weight: 12555.6 ± 5939.5 g). Given the small number of participants tested, the data were analyzed at the level of individual trials using a hierarchical mixed-model using the *lme4 toolbox* in R^10^, with the intercept for participants treated as a random factor and gravity as a fixed factor. P-values were calculated using likelihood ratio tests comparing the model including gravity to the null model. There were clear effects of gravity on weight estimates, both for the hand, χ^2^(1) = 29.31, p < 0.0001, and the head, χ^2^(1) = 29.33, p < 0.0001. To further investigate the quantitative relationship between the strength of physical gravity and perceived body weight, we applied simple linear regressions. Linear regressions were fitted to individual weight estimates in order to determine whether the perceived body-part weight changes linearly across microgravity (0 g), terrestrial gravity (1 g) and hypergravity (1.8 g) conditions. While other patterns (e.g. exponential) might potentially also be present, we had a prior in favour of the simpler, linear pattern. Further, a linear pattern would be consistent with the roughly equi-spaced difference in the strength of physical gravity. Simple linear regressions showed a significant relationship between the strength of physical gravity and perceived weight (all p < 0.01, Fig. 1). The R^2^ values were overall 0.9 confirming that our model explained the 90% of the variability of the response data (S01 Hand: R^2^ = 0.875, Head: R^2^ = 0.892; S02 Hand: R^2^ = 0.931, Head: NA; S03 Hand: R^2^ = 0.941, Head: R^2^ = 0.959). Note that head weight estimates were not obtained for one participant in the microgravity condition due to technical problems.

**Figure 2:**
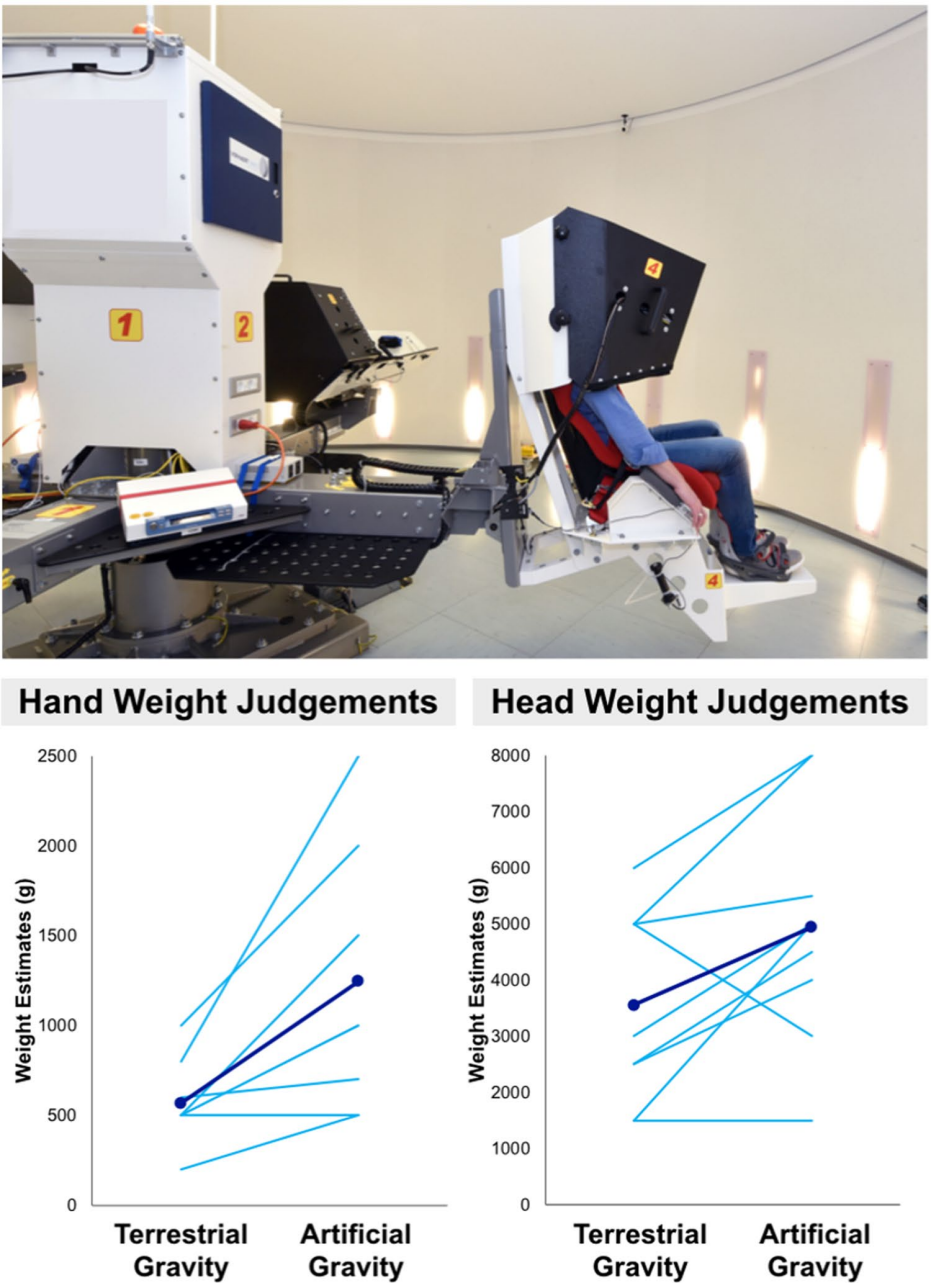
Short arm human centrifuge: Experimental set up and results. Participants were seated on a short arm human centrifuge platform. They made absolute judgements in grams of the perceived weight of their own right hand and head. Weight estimates were collected in normal terrestrial gravity baseline and during +1 g acceleration. Artificial gravity produced an increase in the perceived weight of both the hand and head. Coloured lines reflect data from each individual participant. Blue lines indicate grand averages.

To further investigate the role of gravity in shaping the perceived body weight, we introduced an experimental increase of the experienced strength of gravity using the ESA’s short arm human centrifuge at German Aerospace Center (DLR Cologne), shown in Figure 2. Participants were seated facing outwards on the short arm human centrifuge platform, harnessed within a nacelle. The short arm human centrifuge simulated a +1 g increase in the strength of gravity for ten minutes at head level. As the short arm human centrifuge produces a gradient of centrifugal forces, the increased gravitational force increases progressively towards the participant’s feet (being approximately +1.2 g at their hands). The participant’s head and hand were resting comfortably and they did not have to exert effort to support them. No movements were allowed. Participants were asked to make absolute verbal judgements (in grams) of the perceived weight of their dominant right hand and their head. Weight estimates were collected during a normal terrestrial gravity baseline and during +1 g acceleration, in counterbalanced order.

Participants’ actual weight (*M*: 77.1 ± 11.7 kg) was obtained with a high-precision scale. Based on the results of Tözeren^9^, the actual weight of the hand and the head were estimated as 0.66% and 7.30% of total body weight, respectively. On average, the estimated weight of the hand was 508.9 ± 77.2 g and the weight of the head was 5628.3 ± 853.9 g. Artificial increased gravity produced clear influences on perceived body weight, as seen in Figure 2. There was a clear increase in the perceived weight of both the hand, *t*(8) = 3.75, *p* < 0.01, Cohen’s *d* = 0.34, and the head, *t*(8) = 2.49, *p* < 0.05, Cohen’s *d* = 0.48, during short arm human centrifuge centrifugation compared to a normal terrestrial gravity baseline. To further investigate this effect, we expressed each estimate as a proportion of estimated actual body-part weight. Analysis of variance revealed a significant main effect of gravitational condition, *F*(1, 8) = 22.00, *p* < 0.002, η_p_^2^ = 0.733, with heavier estimates during increased gravity. There was also a significant main effect of body-part, *F*(1, 8) = 20.65, *p* < 0.002, η_p_^2^ = 0.721, with smaller estimates for the head than the hand. There was also a significant interaction of body-part and gravitational condition, *F*(1, 8) = 9.65, *p* < 0.02, η_p_^2^ = 0.547. This interaction showed that the effects of altered gravity were substantially larger for the hand (mean 125% increase, SE 30%) than for the head (mean 24% increase, SE 11%).

## Discussion

The physical weight of our body is determined by the pull of gravity on it. We investigated whether the perceived body weight – like the physical weight – depends on on-line sensory information about the strength of gravity. Participants were asked to estimate the weight of their hand or their head both in normal terrestrial gravity and during exposure to experimentally altered gravitational fields. We found an increase in perceived weight during experience of hypergravity, and a decrease in the perceived weight during experience of microgravity. Our results demonstrate that alterations in the strength of gravitational acceleration produce rapid changes in the perception of body weight. Importantly, participants were not allowed to make any movement and their head and hands were fully supported. Weight estimates therefore relied on the actual sensory information of the strength of gravity and non-sensory specific cues, rather than the experience of effort involved in lifting or supporting body parts.

The central nervous system does not have specialized sensors for the detection of gravity. Rather, gravity is inferred through a process termed *graviception*. The vestibular system senses the pull of gravity via the otolith organs. Otoconia resting atop hair cells are pulled in the direction of gravity when the head moves with respect to the gravitational direction^11–14^. In addition, visual cues, proprioceptive signals from the joints, muscles, skin, and viscera provide references for the body’s position relative to gravity^15^. All these sensory signals are integrated to form an internal model of the magnitude and direction of gravity^13,16^, which are used for visual prediction of object motion^17^. Our results suggest that these models are also used to shape representations of the body itself. Importantly, these estimates are updated by the current strength of gravitational acceleration: the perceived weight of the head and the hand linearly increases with increase in the physical magnitude of gravity.

Regardless of gravitational strength, the perceived weight of the hand was greater as a proportion of actual weight than that of the head. This may reflect differences in the sensory innervation of the two body parts. For instance, the highly innervated skin on the hand may send a strong signal about the contact force between hand and objects, including forces due to the weight of the hand at rest. The difference between hand and head may also relate to known distortions in the perceived size and shape of different body parts^18,19^. The current finding that perceived body weight is rapidly altered by changes in gravitational strength adds to a substantial literature showing that the mental representation of one’s own body is plastic, and sensorially-driven, rather than a fixed semantic fact^20–22^.

Our data show larger individual differences in changes of perceived weight of heads than that of hands. We also found changes in perceived weight of the hand that were greater than those of the head, particularly for the data from the centrifuge. One possibility is that this may reflect the gradient of altered gravity along the body during centrifugation, which was approximately 20% larger for the hands than for the head. However, the magnitude of the perceptual difference between the hand and head was substantially larger than could be explained by this factor alone. Alternatively, this effect may be related to the greater mobility of the hand and its role in skilled actions.

Traditionally, research has focused on the social factors which influence weight perception, as in the putative role of mass media in eating disorders^23^. Misperception of one’s own body weight is a core symptom of anorexia nervosa, and recent research has emphasized its putative role in the “obesity epidemic”^24^. Surprisingly, however, little research has investigated the basic cognitive mechanisms in generating representations of body weight. Our results emphasize the role of basic perceptual mechanisms in shaping the body image. The representation of the body involves an online percept of bodily state, based on the integration of different sensory inputs. On Earth, gravity is *always there*. Although gravitational signals are part of the background of our perceptual world, they play an important role in calibrating the on-line representation of the body. There has been some interest in the sensation of weightlessness experienced by astronauts in outer space, but little formal experimentation. Our results emphasize that the everyday awareness of one’s own body is shaped by ongoing experiences of gravity. The contribution of gravitational signals to this aspect of self-awareness may have been overlooked simply because gravitational signals are ubiquitous, and normally invariant.

## Materials and Methods

### Participants

Following a preliminary medical screening carried out by trained personnel from the German Aerospace Center, Institute of Medicine (DLR Cologne), 3 healthy participants (1 female, 29.7 ± 3.06 years) and 9 healthy participants (3 female, 28.3 ± 5.63 years) provided written informed consent to participate in the parabolic flight study and centrifuge study, respectively. Participants were right handed according to their Edinburgh Handedness Inventory score, and were naive to the purpose of the experiment. The sample size was decided *a priori* based on availability of access to the facilities. The medical examination consisted of a clinical-chemical analysis (glucose, creatinine, urea, uric acid, SGOT, SGPT, γGT, total cholesterol, HDL and LDL), hematology (blood count), urine analysis (glucose, protein, urobilinogen), resting ECG, exercise test to verify endurance capacity, standing test for orthostatic tolerance assessment and medical history. All participants in the parabolic flight had passed the equivalent of an Air Force Class III medical examination. Ethics approval was obtained through the “Ethik-Kommisssion der Ärztekammer Nordrhein” (Ethics Committee of the Regional Medical Association Nordrhein, vote number: 2016086). Procedures were in compliance with German legislation and the Declaration of Helsinki for human participants.

### Parabolic flight procedure

The experiment consisted of a single session. Participants were tested during a European Space Agency (ESA) parabolic flight campaign on board the Airbus Zero-G aircraft (Airbus A310) that took place at the German Aerospace Center, Institute of Medicine (DLR Cologne). During the flight, the aircraft alternated rises (acceleration) and descents (deceleration) to perform the parabolic profiles. Each parabola started with a pull-up phase producing approx. 20 s of hypergravity (+1.8 g), followed by a free-fall phase producing approx. 20 s of microgravity (0 g), and ended with a pull-out phase again producing approx. 20 s of hypergravity (+1.8 g). Baseline measures in terrestrial gravity were performed on board the aircraft before and after the parabolic profiles (flat trajectory at constant velocity). The aircraft performed a sequence of 30 parabolas per flight, and each participant was tested for 15 parabolas.

Participants made absolute verbal judgements (in grams) of the perceived weight of the hand and the head. The order of body-part estimates was counterbalanced across participants. To prevent free floating in the experimental bay, the participants were secured on the plane. The participants were asked to keep their head and hand relaxed, and no movements were allowed. Participants’ actual weight was obtained with a high-precision scale before the flight. Absolute weight judgements were performed in terrestrial (1 g) gravity, microgravity (0 g) and hypergravity (1.8 g) conditions. Head weight estimates were not obtained in the microgravity condition for participant S02 due to technical problems.

### Short arm human centrifuge  procedure

The experiment consisted of a single session. Participants were tested on a short arm human centrifuge at the German Aerospace Center, Institute of Medicine (DLR Cologne). The short arm human centrifuge generates an altered g-environment, producing centrifugal forces exerting an apparent gravitational vector. The centrifugal force produced by the short arm human centrifuge is a function of the square of the angular rate (ω) and the radius (r) of rotation. On Earth, the actual forces exerted on the body during centrifugation are the sum of both standard terrestrial gravity and the additional centrifugal force produced by the rotation. These gravito-inertial forces are larger than, and tilted with respect to, terrestrial gravity. Such altered gravity induces a range of physiological changes, including modulation of signals from the vestibular otoliths which detect the magnitude and direction of gravity^25^.

Participants were harnessed in a nacelle and sat facing outwards on the short arm human centrifuge platform so that their head and torso were perfectly upright with respect to terrestrial gravity. They were seated with knees bent to reduce the distance between the head and feet and hence the gravitational gradient along the body. The interior of the nacelle was dark, preventing vision throughout the experiment.

Participants made absolute verbal judgements (in grams) of the perceived weight of the hand and the head. The order of body-part estimates was counterbalanced across participants. Absolute weight judgements were performed in both terrestrial gravity and artificial gravity conditions. Terrestrial gravity estimates were taken on the centrifuge in the absence of centrifugation. In the artificial gravity condition, the gravitational acceleration was increased to +1 g at head level for a 10 min period, following a standardized acceleration protocol to serve as familiarization. Given the gravitational gradient, the force at the hand is approximately +0.2 g that at the head. Rotation speed was set at 21 rpm and the centrifuge always span clockwise. The weight judgements were taken at the end of the acceleration protocol but during centrifugation at constant velocity. Participants wore a headset with a built-in microphone, which allowed continuous two-way verbal communication with the experimenters. The order of conditions was counterbalanced across participants.

Heart rate was monitored with a 5-lead electrocardiograph (ECG) fitted to the participant, and blood pressure was monitored using a blood pressure cuff combined into a Philips Intellivue X2. Video and audio loops, along with a panic button, were available for the participants’ safety. During the experiment, participants were under continuous visual observation via an infrared camera system monitored by qualified medical personnel and the centrifuge operators. Participants’ heads were fixed securely in a head cushion and their hand on a hand support to prevent movements. Thus, both body parts were resting comfortably and participants did not have to exert effort to support them.
